# Soil Geochemical Controls on Heavy Metal(loid) Accumulation in Tuber Crops from Basalt-Derived Soils and Associated Dietary Intake Health Risks on Hainan Island, China

**DOI:** 10.3390/toxics14010048

**Published:** 2025-12-31

**Authors:** Liling Tang, Jianzhou Yang, Yongwen Cai, Shuqi Hu, Qiuli Gong, Min Zhang, Yong Li, Lei Su

**Affiliations:** 1 State Key Laboratory of Deep Earth and Mineral Exploration, Institute of Geophysical and Geochemical Exploration, Chinese Academy of Geological Sciences, Tianjin 300309, China; tliling@mail.cgs.gov.cn (L.T.); yjianzhou@mail.cgs.gov.cn (J.Y.); hshuqi@mail.cgs.gov.cn (S.H.); liyongigge@outlook.com (Y.L.); 2 The Sixth Geological Brigade of Jiangsu Geological Bureau, Lianyungang 222000, China; zmin1985@outlook.com

**Keywords:** basaltic soil, heavy metal(loid) accumulation, tuber crops, soil geochemistry, nutrient interactions, bioavailability, health risk assessment, volcanic high-background region

## Abstract

Tuber crops cultivated in basalt-derived soils are influenced by naturally high geochemical backgrounds, which may elevate heavy metal(loid) levels and associated health risks. To clarify the geochemical controls governing metal accumulation, this study analyzed rock, soil, and tuber (sweet potato and yam) samples from the Qiongbei volcanic area of Hainan Island, China. Concentrations of eight heavy metal(loid)s (As, Cd, Cr, Cu, Hg, Ni, Pb, and Zn) and 22 nutrient-related indicators (N, P, K, SOC, S, Se, Fe, Mn, and their available fractions) were determined. Soil contamination and potential human health risks were evaluated using the pollution index and the health risk model. The results showed that 11.1–55.6% of soil samples exceeded pollution thresholds for Cr, Cu, Ni, and Zn, reflecting typical basaltic high-background characteristics. In contrast, heavy metal(loid) concentrations in tuber crops were relatively low and jointly regulated by parent material composition and soil nutrient status. Non-carcinogenic risks (HI) were below 1, indicating acceptable exposure levels, while carcinogenic risks were mainly associated with Cd, Cr, and Pb, with total carcinogenic risk (TCR) exceeding 1 × 10^−4^, suggesting potential health concerns. Strong correlations between soil nutrients (N, P, K, SOC, S, Se, Mn, and Fe) and plant uptake of As, Cd, Cu, and Cr indicate that nutrient availability plays a crucial role in controlling heavy metal(loid) bioavailability. The volcanic soils exhibited a “high total content–low bioavailability” pattern. Enhancing soil Se, SOC, available N, and slowly available K (SAK) can effectively reduce Cd and other high-risk metal accumulation in tuber crops. These findings elucidate the key geochemical processes influencing heavy metal transfer in volcanic agroecosystems and provide a scientific basis for safe agricultural utilization and health risk prevention in high-background regions.

## 1. Introduction

Tuber crops such as sweet potato (Ipomoea batatas) and yam (Dioscorea spp.) are staple foods for over 60 millions of people worldwide, particularly in tropical and subtropical regions [1]. They not only provide a major source of carbohydrates but are also rich in vitamins, minerals, and trace nutrients, playing an essential role in food security and human nutrition [2]. However, increasing environmental pollution, combined with naturally high geochemical backgrounds in certain regions, has raised concerns about the accumulation of potentially toxic heavy metals in agricultural products [3]. Because many heavy metals are persistent, bioaccumulative, and non-degradable, their entry into the food chain can cause neurological disorders, hepatic and renal dysfunction, and metabolic abnormalities [4]. Unlike grains or leafy vegetables, tuber crops grow underground in direct contact with soil, making them more susceptible to heavy metal uptake and accumulation [5]. Therefore, understanding the migration and enrichment of heavy metals within soil–tuber systems is of great importance for ensuring food safety and assessing potential health risks.

Total soil metal concentrations are widely used for contamination screening, yet they often fail to accurately reflect the fractions that are bioavailable to plants [6]. The geochemical behavior and bioavailability of heavy metals in soil are controlled by a combination of physicochemical factors, including pH, organic matter content, mineral composition, redox conditions, and nutrient status [7,8]. In volcanic high-background regions, the parent materials, typically basalts, are naturally enriched in Cr, Ni, and Cu [9]. Nevertheless, high total concentrations do not necessarily imply high bioavailability [10]. Processes such as adsorption, complexation, and ion competition can immobilize metals on mineral surfaces or organic complexes, thereby limiting plant uptake [11,12]. Disentangling total and bioavailable metal fractions is therefore a key step toward accurately evaluating soil–crop interactions and food safety in high-background areas.

In recent years, research on heavy metal transfer in soil–plant systems and associated dietary exposure risks has expanded considerably. For example, Xiao et al. (2014) [13] reported that Pb concentrations were generally higher than Cd concentrations across crop species in Hainan Island, highlighting soil elemental composition and crop type as key controls on metal accumulation. Similarly, Yang et al. (2021) [14] found that dietary exposure risks from leafy vegetables were significantly higher than those from tuber crops and other non-leafy vegetables in the same region. However, most studies have focused on rice, wheat, and leafy vegetables, while investigations on tuber crops remain limited [15,16,17]. Moreover, many existing studies rely on controlled laboratory experiments, which may not capture the complex geochemical processes and spatial heterogeneity of natural farmlands [18]. This is particularly true in tropical volcanic regions, where soil formation is governed by intense weathering, high rainfall, and rapid organic matter cycling, creating pronounced spatial and temporal dynamics [18]. Yet, the mechanisms governing heavy metal accumulation in tuber crops and the related human health risks under these conditions remain poorly understood. Health risk assessment models provide a valuable quantitative framework for linking environmental geochemistry to human exposure. The U.S. Environmental Protection Agency (USEPA) framework, which includes the non-carcinogenic risk indices (HQ and HI) and the carcinogenic risk index (CR) [19], is widely used to evaluate human health risks from heavy metal ingestion through food. Applying these models to geochemical high-background regions can help distinguish natural enrichment from anthropogenic inputs and offer a scientific basis for developing dietary safety guidelines and pollution management strategies.

Hainan Island, located in southern China, contains extensive Cenozoic basalt formations, particularly in its northern region. Long-term tropical weathering of these basalts has produced lateritic soils rich in Cr, Ni, Cu, and Zn [20]. The region experiences minimal industrial contamination but widespread agricultural activity [21], providing an ideal natural laboratory for distinguishing between parent-material and anthropogenic influences. Sweet potato is a major local staple crop and an important source of dietary energy and nutrients for residents, making it a representative model for studying the coupled processes of “rock–soil–crop–human health” in volcanic environments. This study focuses on the Qiongbei volcanic region of Hainan Island to elucidate the geochemical mechanisms of heavy metal(loid) accumulation in tuber crops and their potential health implications. Specifically, the objectives are to: (1) characterize the physicochemical and nutrient properties of soils, including N, P, K, pH, SOC, and available nutrient fractions; (2) quantify heavy metal(loid) concentrations and distribution patterns in the rock–soil–tuber continuum; (3) identify the key soil factors controlling metal bioavailability and crop accumulation; and (4) assess the non-carcinogenic and carcinogenic health risks associated with metal intake through sweet potato consumption based on local dietary patterns. By integrating geochemical analyses, nutrient interactions, and human health risk assessment, this study aims to reveal the processes governing heavy metal(loid) transfer in volcanic high-background agroecosystems and to provide a scientific foundation for ensuring food safety, improving nutritional security, and promoting sustainable soil management in tropical regions.

## 2. Materials and Methods

### 2.1. Study Area

The study area is situated in the volcanic platform region of northern Hainan Island, southern China, which represents a typical cultivation zone for sweet potato and Chinese yam. The region experiences a tropical monsoon climate characterized by a mean annual temperature of 24.0 °C and average annual precipitation of 1786.1 mm [22]. Rainfall and temperature are seasonally synchronized, with distinct wet and dry periods, and frost-free conditions prevail year-round.

The dominant soil type is lateritic red soil (tropical ferrallitic soil) derived from Cenozoic basalt, with land use primarily devoted to agriculture—particularly the cultivation of tuber crops. Soils are predominantly clay loam in texture, with subordinate loam and clay. The clay fraction ranges from 24 to 36% (average 28%), silt from 0 to 20% (average 6%), and sand from 44 to 72% (average 67%) [22]. The area has a long history of sweet potato and yam cultivation; these crops are important for both local consumption and export. Ridge planting is commonly practiced, with a planting density of approximately 0.15 m × 0.80 m.

### 2.2. Sample Collection

**Figure 1:**
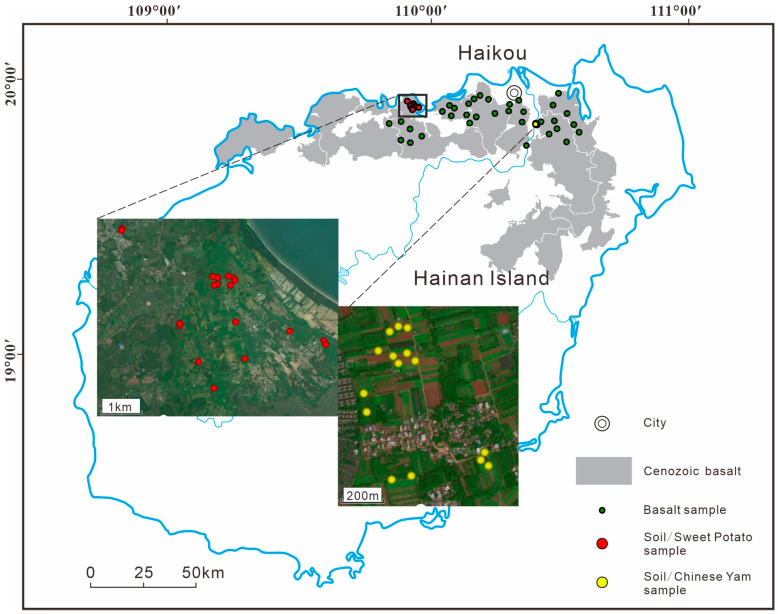
Location of the study area and distribution of sampling sites.

Basaltic bedrock samples were collected from outcrops distributed around the major cultivation zones and were evenly spaced across the basaltic region as far as possible to enhance representativeness. A total of 34 basalt samples were obtained to represent the parent material of the local soils (Figure 1). Plant and corresponding rhizosphere soil samples were collected from the core cultivation areas of tuber crops during the maturity stage. Sampling density was determined according to plantation area: 1–3 samples were collected from plantations smaller than 30 mu, while for plantations larger than 30 mu, one sample was collected for every 10–20 mu. In total, 20 sweet potato and 15 yam samples were collected (Figure 1). Each composite plant sample consisted of 3–5 individual plants (five for sweet potato and three for yam) collected within an area of approximately 100 m^2^, with a total fresh weight of 3–5 kg.

For each plant sample, the corresponding rhizosphere soil was collected from the 0–20 cm plow layer around the root zone. Soil samples corresponded one-to-one with plant samples, and subsamples were thoroughly mixed to form a composite sample, with each final sample weighing no less than 1 kg. Stones, roots, and plant debris were removed. The soils were homogenized, air-dried, and passed through a 2 mm sieve for subsequent geochemical and nutrient analyses.

### 2.3. Sample Preparation and Analytical Methods

Plant analyses were conducted at the Hunan Institute of Geological Testing. Tuber samples were peeled with a ceramic knife, chopped into small pieces, and weighed. The subsamples were oven-dried at 60 °C to a constant weight, after which moisture content was determined. Dried samples were ground into fine powder using a high-speed pulverizer. For digestion, 0.5 g of powdered material was placed in a microwave digestion vessel and mixed with 3–5 mL of HNO_3_. After standing for 1 h, the vessel was sealed and digested. Following cooling and venting, the vessel was rinsed with deionized water, and the solution was further heated on a thermostatic plate at 100 °C for 30 min. The final digests were diluted to 25 mL or 50 mL with deionized water, homogenized, and analyzed. Concentrations of eight heavy metals (As, Cd, Cr, Cu, Ni, Pb, Zn, and Hg) were determined using inductively coupled plasma mass spectrometry (ICP–MS). Analytical quality was monitored using certified reference materials GBW10011 [23] and GBW10048 [24]. The relative errors for Cr and Hg were better than 8.67%, while those for the remaining metals were better than 5%.

Rock and soil sample analyses were carried out at the Institute of Geophysical and Geochemical Exploration, Chinese Academy of Geological Sciences. Rock samples were cleaned, air-dried, and ground to <0.074 mm prior to analysis. Detailed pretreatment, analytical procedures, and quality control protocols followed the methods described by [25] and the DZ/T 0258–2014 standard [26]. Analytical techniques were selected based on the target parameters, and the detection limits for all analyzed indicators are summarized in Table 1. Quality assurance and quality control (QA/QC) were maintained through the use of standard reference materials, procedural blanks, and replicate measurements. The certified reference material GBW07390 [27] was used for rock samples, GSS30 [28] for soil samples, and AR-1 and AR-6 for soil available fractions. The calculated relative errors and relative standard deviations for all analytical results were within the acceptable limits specified by the DZ/T 0258–2014 standard [26].

### 2.4. Pollution Assessment Methods

To evaluate the contamination levels of heavy metals in soils, both the single-factor pollution index (*P_i_*) and the Nemerow comprehensive pollution index (*P*_N_) were applied [29]. Their calculation formulas are as follows:

(1) $$P_{i}\;=\;\frac{C_{i}}{S_{i}}$$

(2) $$P_{N}=\sqrt{\frac{{\left(\bar{P_{i}}\right)}^{2}+{\left(P_{max}\right)}^{2}}{2}}$$

where *P_i_* is the single-factor pollution index of heavy metal *i*; *C_i_* and *S_i_* represent the measured concentration and the corresponding standard limit for element *i*, respectively. *P*_N_ is the Nemerow comprehensive pollution index; *P*_max_ is the maximum single-factor pollution index among all metals, and P_ave_ is their average value.

The pollution levels were classified as follows: Single-factor pollution index (*P_i_*): No pollution (*P_i_* ≤ 0.7); Precaution level (0.7 < *P_i_* ≤ 1.0); Slight pollution (1.0 < *P_i_* ≤ 2.0); Moderate pollution (2.0 < *P_i_* ≤ 3.0); Heavy pollution (*P_i_* > 3.0). Comprehensive pollution index (*P*_N_): No pollution (*P*_N_ ≤ 0.7); Precaution level (0.7 < *P*_N_ ≤ 1.0); Slight pollution (1.0 < *P*_N_ ≤ 2.0); Moderate pollution (2.0 < *P*_N_ ≤ 3.0); Heavy pollution (*P*_N_ > 3.0) [30].

### 2.5. Health Risk Assessment Methods

To assess potential human health risks associated with the consumption of tuber crops contaminated by heavy metals, both non-carcinogenic and carcinogenic risk models were adopted based on the U.S. Environmental Protection Agency (USEPA) methodology.

#### 2.5.1. Non-Carcinogenic Risk

Non-carcinogenic risk was characterized by the hazard quotient (HQ) and the hazard index (HI) [31], calculated as:

(3) $$\mathrm{HQ}\;=\frac{\mathrm{EDI}}{RfD}=\frac{C\;\times \;\mathrm{IR}\;\times \;\mathrm{EF}\;\times \;\mathrm{ED}}{BW\times \;\mathrm{AT}\;\times \;RfD}$$

(4) $$\mathrm{HI}=\sum_{i=0}^{n}{HQ}_{i}$$

where EDI is the estimated daily intake of heavy metal; C is the metal concentration in the crop (mg·kg^−1^); IR is the average ingestion rate (0.075 kg·day^−1^) [32]; EF, ED, BW, and AT denote exposure frequency (350 days·year^−1^), exposure duration (70 years), average body weight (60.3 kg), and averaging time (25,550 days), respectively [33]. RfD represents the oral reference dose (mg·kg^−1^·day^−1^), with the following values: As 0.0003, Cd 0.001, Cr 0.003, Cu 0.04, Hg 0.0003, Ni 0.02, Pb 0.004, and Zn 0.3 [19]. When HQ or HI < 1, the exposure level is considered acceptable, whereas HQ or HI > 1 indicates a potential non-carcinogenic health risk.

#### 2.5.2. Carcinogenic Risk

Carcinogenic risk was expressed using the cancer risk coefficient (CR), calculated as:

CR = EDI × CF(5)

(6) $$\mathrm{TCR}=\sum_{i=1}^{5}CR$$

where CF is the cancer slope factor (mg·kg^−1^·day^−1^)^−1^. In this study, As, Cd, Cr, Ni, and Pb were considered the primary carcinogenic elements, with CF values of 1.5, 15, 0.5, 0.0085, and 1.7, respectively [16]. According to the USEPA standard, CR < 1 × 10^−4^ indicates an acceptable risk level, whereas CR > 1 × 10^−4^ suggests a potential lifetime carcinogenic risk.

## 3. Results

### 3.1. Soil Physicochemical Composition

#### Distribution and Pollution Characteristics of Soil Nutrients and Heavy Metal(loid)s

Soil pH in the study area ranged from 4.6 to 7.7, with an average of 5.4 (Table 2), indicating generally weakly acidic to neutral conditions. The average SOC content was 0.81% (Table 2), reaching up to 1.42% in some samples, reflecting an overall low level of organic matter. Major nutrient elements showed substantial variability: total N averaged 809 mg/kg (Table 2), with some sites exceeding 1500 mg/kg, suggesting pronounced spatial heterogeneity; total P averaged 955 mg/kg (Table 2), representing a moderate level; K_2_O ranged from 0.05% to 0.35% (mean 0.19%, Table 2), indicating a relatively low potassium status; and total sulfur (S) averaged 441 mg/kg.

For available nutrient fractions, the mean concentrations of hydrolyzable N, available P, readily available K, slowly available K, exchangeable Na, exchangeable Ca, exchangeable Mn, easily reducible Mn, available Mn, available Cu, available Zn, available Fe, and available S were 84.26, 91.74, 211.61, 91.77, 21.24, 368.21, 40.14, 344.75, 61.54, 1.82, 2.91, 187.85, and 225.56 mg/kg, respectively (Table 2), indicating significant differences in the supply capacity among nutrient components.

Coefficient of variation (CV) analysis showed that soil pH and hydrolyzable N exhibited low (<15%) and moderate (15–35%) variability, respectively, while the other nutrients displayed strong variability (CV > 35%) (Table 2). This pattern suggests considerable spatial heterogeneity in soil nutrient availability and effective fractions across the volcanic area, likely related to differences in parent material composition, fertilization management, and crop rotation practices.

### 3.2. Heavy Metal(loid) Concentrations in Rocks, Soils, and Tuber Crops

**Figure 2:**
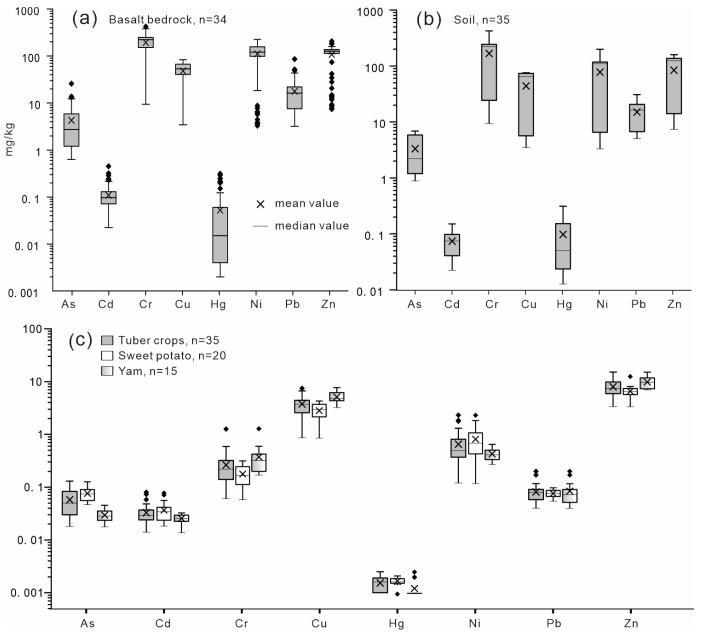
Heavy metal(loid) concentrations in (**a**) basalt bedrock, (**b**) soil, and (**c**) tuber crops.

**Figure 3:**
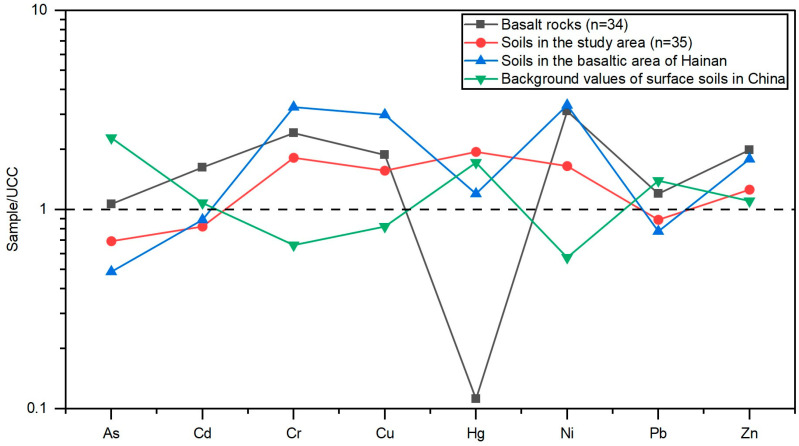
Distribution characteristics of heavy metal(loid)s in basalt and soil normalized to the UCC. The dashed line represents the composition of the upper continental crust (UCC), after Rudnick and Gao (2014) [34].

Heavy metal(loid) concentrations in basalt, soil, and tuber crops are illustrated in Figure 2. Both basaltic bedrock and corresponding soils contained high levels of Cr, Cu, Ni, and Zn, whereas As, Cd, Hg, and Pb concentrations were relatively low. The mean concentrations of heavy metal(loid)s in basalt followed the order Cr (222.4 mg/kg) > Ni (146.4 mg/kg) > Zn (133.6 mg/kg) > Cu (52.7 mg/kg) > Pb (20.4 mg/kg) > As (5.11 mg/kg) > Cd (0.15 mg/kg) > Hg (5.59 μg/kg), comparable to average background values for Chinese basalts. Compared with the upper continental crust (UCC), the studied basalts showed enrichment in all heavy metal(loid)s except Hg, which was markedly depleted (Figure 3).

In soils, mean concentrations of As, Cd, Cr, Cu, Hg, Ni, Pb, and Zn were 3.33 mg/kg, 73.98 μg/kg, 167.42 mg/kg, 43.88 mg/kg, 97.39 μg/kg, 77.77 mg/kg, 15.09 mg/kg, and 84.31 mg/kg, respectively, consistent with the high-background geochemical characteristics of basaltic terrains. Relative to the Chinese surface soil background, Cr, Ni, and Cu were notably enriched (Figure 3). Tuber crops (sweet potato and yam) also exhibited elevated levels of Cr, Cu, Ni, and Zn compared with other heavy metal(loid)s, reflecting an inherited geochemical signature from the parent material and soil.

**Figure 4:**
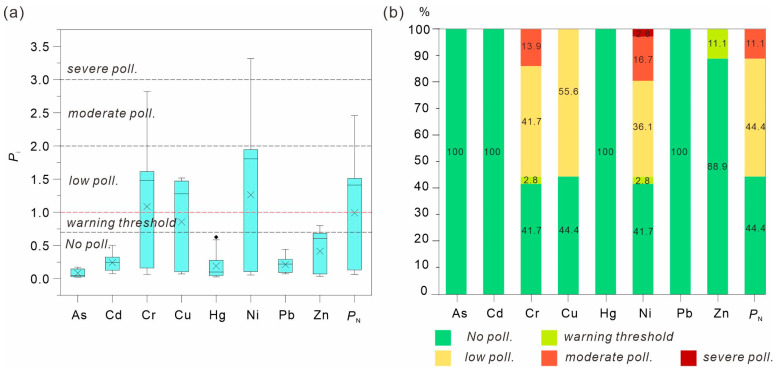
Pollution levels of heavy metal(loid)s in the soil of the study area. (**a**) Pollution Distribution; (**b**) Proportion of Different Pollution Levels.

Given that the local soils are generally weakly acidic (Table 2), the most stringent soil contamination screening values were applied (As 40 mg/kg, Cd 300 μg/kg, Cr 150 mg/kg, Cu 50 mg/kg, Hg 500 μg/kg, Ni 60 mg/kg, Pb 70 mg/kg, Zn 200 mg/kg, [35]). The average Nemerow integrated pollution index (*P*_N_) was 1.0, indicating that the majority of soils were unpolluted to slightly polluted, accounting for 44.4% of samples, with 11.1% showing moderate pollution levels (Figure 4). According to the single-factor pollution index (*P_i_*), Cr, Ni, and Cu were identified as the dominant potential pollutants, as more than half of the samples (55.6%) exceeded light pollution thresholds for these elements, while other metals remained within safe limits (Figure 4). Overall, the spatial distribution of heavy metal(loid)s closely mirrored that of the basaltic parent material, suggesting that soil enrichment primarily reflects geological background rather than anthropogenic input.

For tuber crops, the mean concentrations of As, Cd, Hg, and Ni in sweet potato were generally higher than those observed in yam, although the overall levels of the eight analyzed heavy metal(loid)s were comparable between the two tuber species (Figure 2c). According to the National Food Safety Standard for Maximum Levels of Contaminants in Foods (GB 2762–2017) [36], the permissible limits for Cu, Pb, Zn, Cr, Cd, As, and Hg are 10, 0.2, 20, 0.5, 0.1, 0.5, and 0.01 mg/kg, respectively. While no national guideline has been established for Ni in tuber crops, approximately 13.9% of the samples exceeded the 1 mg/kg dry-weight threshold proposed by [16]. However, after adjusting for the high moisture content of tubers, all samples met the safety standards on a fresh-weight basis. Thus, the overall edible safety of tuber crops in the study area is considered acceptable. Nevertheless, as these crops constitute a dietary staple for local residents, long-term exposure risks warrant further assessment through dietary intake and health risk modeling.

### 3.3. Health Risks to Residents from Dietary Intake of Tuber Crops

According to the National Food Safety Standards of China (GB 2762–2017) [36], the concentrations of eight heavy metal(loid)s in tuber crops from the study area were generally below the permissible limits, with only a few samples showing slightly elevated Ni levels relative to the reference threshold. Nevertheless, since tuber crops represent a major energy source in the local diet, long-term and continuous consumption may still pose potential health risks.

**Figure 5:**
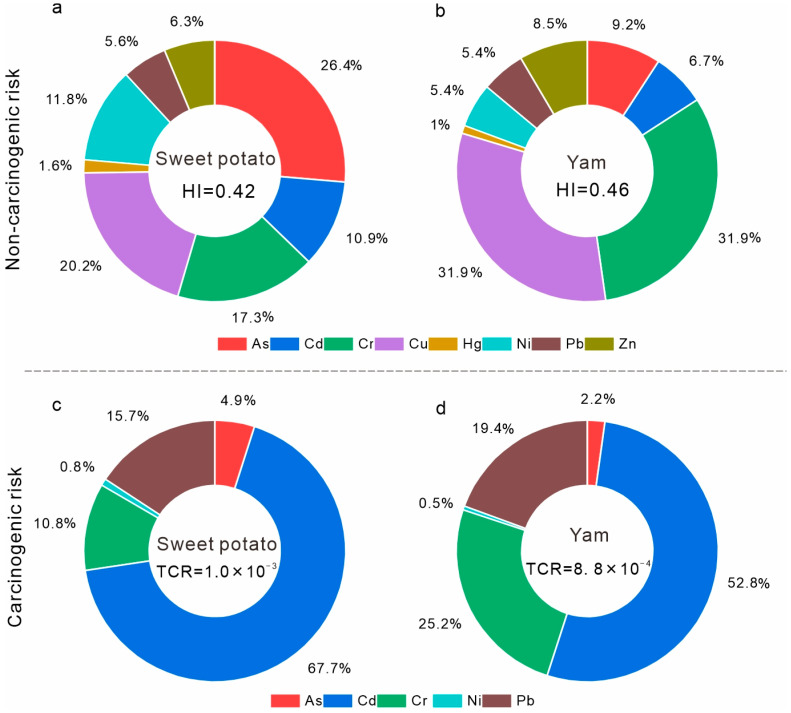
Health risks from heavy metal(loid) consumption in tuber crops: (**a**,**b**) non-carcinogenic risks of sweet potato and Chinese yam, respectively; (**c**,**d**) carcinogenic risks of sweet potato and Chinese yam, respectively.

Results of the non-carcinogenic risk assessment indicated that the hazard index (HI) values for sweet potatoes and yams were 0.42 and 0.46, respectively, with an average of 0.44 (Figure 5a,b). All values were below the safety threshold of 1, suggesting that heavy metal(loid) exposure through tuber consumption poses no significant non-carcinogenic risk to local residents. Arsenic, Cr and Cu were identified as the dominant contributors to non-carcinogenic risk, jointly accounting for more than 73% of the total HI (Figure 5a,b), although their combined effect remained within the acceptable range.

The results of the carcinogenic risk assessment indicated that the total carcinogenic risk (TCR) values for sweet potatoes and yams were 1.0 × 10^−3^ and 8.8 × 10^−4^, respectively, with an average value of 9.4 × 10^−4^ (Figure 5c,d). Overall, both tuber species exhibited comparable levels of potential carcinogenic risk. The individual carcinogenic risk (CR) values of As and Ni in both sweet potatoes and yams were well below the acceptable threshold of 1 × 10^−4^, suggesting negligible carcinogenic concern. In contrast, Cd, Cr, and Pb showed slightly elevated CR values exceeding the USEPA guideline (1 × 10^−4^). Specifically, the CR values of Cd, Cr, and Pb were 6.97 × 10^−4^, 1.08 × 10^−4^, and 1.58 × 10^−4^ for sweet potatoes, and 4.66 × 10^−4^, 2.22 × 10^−4^, and 1.71 × 10^−4^ for yams, respectively, indicating a potential carcinogenic risk. These three elements collectively contributed more than 90% of the TCR, with Cd identified as the dominant contributor, accounting for 67.7% and 52.8% of the TCR in sweet potatoes and yams, respectively (Figure 5c,d).

Notably, although the non-carcinogenic risk associated with tuber crop consumption in this study remains within the acceptable range, the total carcinogenic risk substantially exceeds the recommended threshold, which may appear contradictory. However, this phenomenon is not unique to tuber crops. For example, Wang et al. (2020) [16] reported that the hazard index (HI) for wheat consumption also indicated acceptable safety, whereas the total carcinogenic risk (TCR) reached 1.08 × 10^−3^. This discrepancy arises from fundamental differences in the calculation frameworks of HI and TCR. The HI is primarily determined by metal concentrations in crops relative to their respective reference doses, whereas TCR additionally incorporates daily intake rates, metal-specific carcinogenic slope factors, and the number of carcinogenic metals considered. Consequently, TCR is more sensitive to cumulative exposure effects. These results suggest that the current health risk assessment framework may require further refinement. In particular, while a uniform threshold of 10^−4^ is commonly applied to assess carcinogenic risk for both single metals and multiple metals combined, adjusting the threshold to account for the number of carcinogenic elements considered may provide a more realistic evaluation of cumulative cancer risk.

## 4. Discussion

### 4.1. Variations in Heavy Metal(loid) Contents During Basalt Weathering and Soil Formation

**Figure 6:**
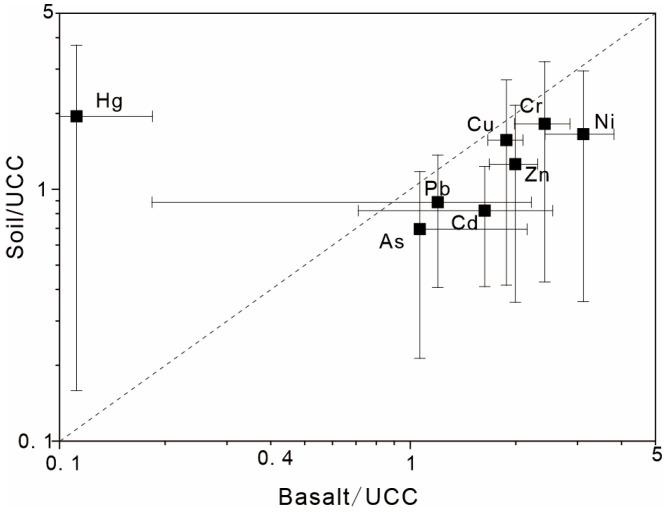
Ratios of heavy metal concentrations in the basalt bedrock and topsoil of the study area normalized to the UCC [34].

To elucidate the geochemical evolution of heavy metal(loid)s during basalt weathering and soil formation, concentrations of heavy metal(loid)s in basalts and their derived soils were normalized to the UCC [34] (Figure 6). The results showed that, relative to the UCC, basalts were enriched in seven heavy metal(loid)s (As, Cd, Cr, Cu, Ni, Pb, Zn) but depleted in Hg, with particularly high enrichments of Cr, Cu, Ni, and Zn—approximately twice the UCC levels (Figure 3). In contrast, soils developed from basalts exhibited enrichment in Cr, Cu, Hg, Ni, and Zn, but relative depletion in As, Cd, and Pb. This pattern is consistent with previous findings for basaltic soils in Hainan Island [20], indicating that the geochemical signature of the parent basalt exerts a dominant influence on soil heavy metal(loid) composition.

Heavy metal(loid)s, except for Hg, were lower in soils than in basalts (Figure 3), suggesting partial leaching losses during weathering. In contrast, soil Hg concentrations exceeded those of the parent basalt, with normalized values plotting above the 1:1 line (Figure 6), reflecting distinct enrichment during pedogenesis. This enrichment is likely attributable to exogenous inputs, such as atmospheric deposition or agricultural activities [30,37], rather than lithogenic inheritance. Conversely, the other seven heavy metal(loid)s plot below the 1:1 line (Figure 6), indicating that they are primarily derived from the parent basalt but have undergone minor depletion during soil formation. Overall, except for Hg, heavy metal(loid)s in the study area’s soils largely inherit the basaltic geochemical characteristics, with moderate migration and loss accompanying pedogenic processes.

### 4.2. Influence of Soil Heavy Metal(loid)s on Metal Accumulation in Tuber Crops

**Figure 7:**
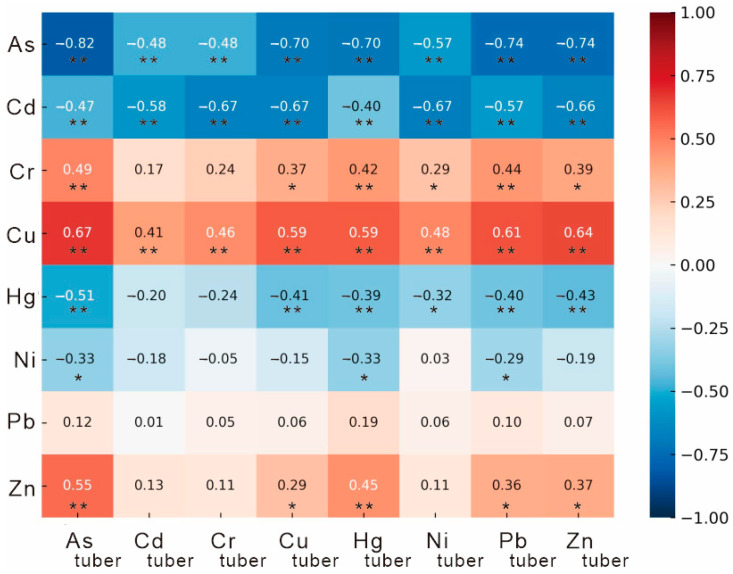
Correlation matrix between heavy metal(loid)s in soil and tuber crops. **, *p* < 0.01; *, *p* < 0.05.

Soil acts as the primary source of heavy metal(loid) uptake and accumulation in plants. Correlation analyses between rhizosphere soils and tuber crops revealed a significant positive relationship between Cu concentrations in soils and those in tubers, with Cr, Zn, and Pb also showing moderate positive correlations (Figure 7). In contrast, As, Cd, and Hg exhibited negative correlations with their soil counterparts (Figure 7). These results indicate that tuber crops respond differentially to various heavy metal(loid)s, with selective uptake and exclusion mechanisms influencing their accumulation behavior.

Overall, only Cu, Cr, and Zn showed consistent enrichment trends between soils and tubers, while As, Cd, and Hg displayed inverse relationships (Figure 7), suggesting that their bioavailability in the rhizosphere is restricted. This finding highlights that high total concentrations of heavy metal(loid)s in volcanic soils do not necessarily translate into elevated levels in edible plant tissues. The abundance of Fe–Mn oxides, clay minerals, and organic matter formed during basalt weathering can effectively immobilize metal ions through adsorption, complexation, and co-precipitation processes [7,8]. Consequently, many metals exist in a “high total–low available” state, markedly reducing their bioavailability and limiting their translocation to crop tissues [10]. Thus, despite the elevated total concentrations of certain metals in the soils, their limited mobility and bioavailability result in relatively low accumulation levels in tuber crops. This “high-background–low-bioavailability” characteristic constitutes the geochemical foundation for the observed food safety of tuber crops cultivated in the basaltic regions of northern Hainan Island.

### 4.3. Soil Nutrients and Their Available Fractions Influencing Heavy-Metal Uptake in Tuber Crops

**Figure 8:**
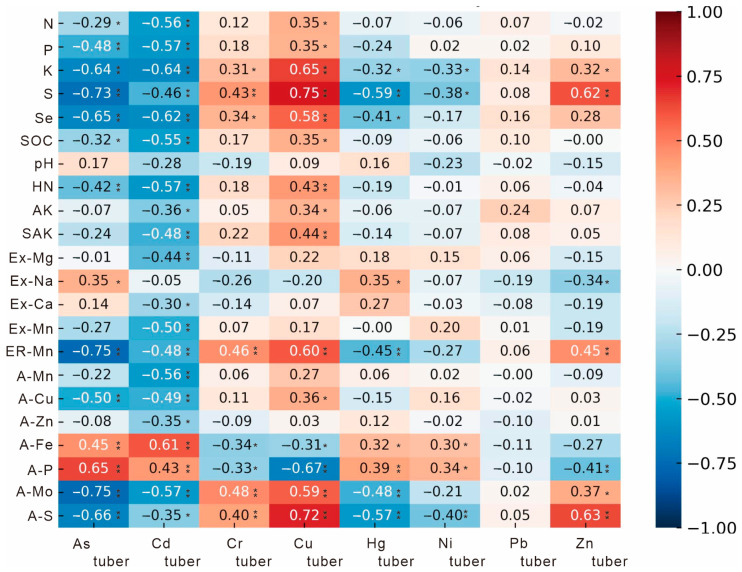
Correlation matrix between soil physicochemical properties and heavy metal(loid)s in tuber crops. **, *p* < 0.01; *, *p* < 0.05.

Plant uptake of heavy metal(loid)s is rarely governed by a simple linear relationship with the total concentration of an element in soil; rather, it is controlled by multiple physico-chemical factors of the soil–plant system [7,8]. In the present study, we analyzed the correlations between 22 soil indicators (including N, P, K, pH, SOC, and their labile/available fractions) and heavy-metal concentrations in tuber crops (Figure 8). The results demonstrate that the uptake of different metals is governed by distinct nutrient and redox-sensitive pathways.

For arsenic in tubers, we observed significant negative correlations with most soil nutrient indices—notably S (r = –0.73, *p* < 0.01), Se (r = –0.65, *p* < 0.01) and ERR-Mn (r = –0.75, *p* < 0.01) (Figure 8). This suggests that higher soil S, Se and labile Mn may suppress As uptake, possibly because of competitive adsorption or co-precipitation of sulfate/selenate anions on Mn oxides, thereby reducing As bioavailability [38,39,40]. In addition, As was strongly negatively correlated with A-Mo (r = –0.75, *p* < 0.01) and available S (r = –0.66, *p* < 0.01), indicating that a soil environment with stronger redox activity favors As immobilization. In contrast, positive correlations of As with AP (r = 0.65, *p* < 0.01) and A-Fe (r = 0.45, *p* < 0.01) reflect the dual role of phosphate and Fe–oxides in modulating As bioavailability—on one hand promoting adsorption, on the other affecting release via ligand exchange [41]. These findings align with studies showing that soil redox and nutrient dynamics jointly regulate As mobility [41,42].

Regarding cadmium, tuber Cd concentrations were largely negatively correlated with most nutrient indices (particularly N, P, K; r < –0.55, *p* < 0.01), suggesting that higher fertility may reduce Cd accumulation via a “dilution effect” or by promoting greater biomass which lowers metal concentration per unit mass [43,44]. A negative correlation with Se (r = –0.62, *p* < 0.01), SOC (r = –0.55, *p* < 0.01) and Ex Mn (r = –0.50, *p* < 0.01) implies that organic matter complexation and Mn oxide adsorption are key mechanisms for Cd immobilization [45,46]. Typically, Fe and Mn oxides adsorb Cd and inhibit plant uptake [47,48]; however, an unexpected positive correlation was observed between Cd and available Fe (r = 0.61, *p* < 0.01). This relationship may reflect conditions under which Fe oxides enhance Cd mobilization, for example through competitive interactions or reductive dissolution processes. The weak correlation between Cd and pH suggests that mineralogical controls and nutrient status exert a stronger influence on Cd availability than pH alone.

For chromium, tuber Cr concentrations exhibited significant positive correlations with soil S (r = 0.43, *p* < 0.01), Se (r = 0.34, *p* < 0.05), and available Mo (r = 0.48, *p* < 0.01) (Figure 8). These relationships suggest that sulfur- and selenium-rich environments may facilitate the formation of Cr–organic complexes or enhance ligand-mediated Cr mobility, thereby increasing bioavailability [49,50]. Notably, studies on As-hyperaccumulating species have also shown that Se application can, under certain conditions, promote Cr uptake while simultaneously alleviating Cr toxicity [49], implying that Se-mediated enhancement of Cr bioavailability may be accompanied by physiological detoxification mechanisms. The positive correlation with reducible Mn (r = 0.46, *p* < 0.01) further indicates that Mn redox cycling influences Cr speciation and plant uptake, whereas the slight negative correlation with available Fe (r = –0.34, *p* < 0.05) suggests that Fe oxides may act as sinks constraining Cr acquisition by roots. Considering that basalt-derived soils in our study area tend to have high Cr content, increasing available Fe and P may help reduce Cr uptake in tubers.

For copper, its tuber concentration was strongly positively correlated with S (r = 0.75, *p* < 0.01), AS (r = 0.72, *p* < 0.01) and ERR-Mn (r = 0.60, *p* < 0.01) (Figure 8). These results indicate that sulfur-rich and redox-active conditions enhance Cu accumulation, likely because Cu readily forms soluble complexes with sulfur-ligands, facilitating plant uptake [50,51]. Negative correlations with AP (r = –0.67, *p* < 0.01) and A-Fe (r = –0.31, *p* < 0.05) point to the inhibitory influence of phosphate and Fe oxides via adsorption or precipitation, thereby reducing Cu availability. Such nutrient–metal interactions have been reported in various plant–soil systems [51,52].

For mercury, tuber Hg content was negatively correlated with K (r = –0.32, *p* < 0.05), S (r = –0.59, *p* < 0.01) and AS (r = –0.57, *p* < 0.01) (Figure 8), implying that elevated K and S levels may reduce Hg uptake, perhaps via competition at sorption sites or enhanced Hg–organic-matter complexation. A weak positive correlation with AP (r = 0.39, *p* < 0.01) suggests that phosphate may promote Hg mobilization through competitive adsorption. Although the present data indicate that Hg bioavailability appears to be jointly regulated by nutrient status and soil anion composition, a substantial proportion of plant-accumulated Hg often originates from atmospheric deposition rather than root uptake [53,54], and the mechanisms governing Hg assimilation in plants warrant further investigation.

Nickel exhibited generally weak correlations with most soil indicators; only AS (r = –0.40, *p* < 0.01), S (r = –0.38, *p* < 0.05), and K (r = –0.33, *p* < 0.05) showed modest negative relationships (Figure 8). This suggests that Ni uptake in tubers is primarily governed by soil background concentrations, while higher levels of S and K may inhibit Ni absorption. Under field conditions, appropriate supplementation of these nutrients could potentially reduce Ni accumulation in plants growing in volcanic regions with high natural Ni backgrounds.

Lead exhibited no significant correlations with soil nutrients (Figure 8), indicating that its accumulation is primarily governed by geogenic background rather than nutrient status. Given Pb’s strong soil binding and limited translocation in plants, nutrient management alone is unlikely to affect its uptake.

For zinc, tuber Zn concentrations exhibited significant positive correlations with AS (r = 0.63, *p* < 0.01), S (r = 0.62, *p* < 0.01), and ERR-Mn (r = 0.45, *p* < 0.01), indicating that S-rich and nutrient-enriched environments may enhance Zn solubility and plant availability [55]. In contrast, negative correlations with AP (r = –0.41, *p* < 0.01) and Ex-Na (r = –0.34, *p* < 0.05) indicate that excessive phosphate or elevated ionic strength (associated with exchangeable Na) may suppress Zn uptake, likely due to the formation of insoluble Zn–phosphate complexes or ion-strength effects that reduce Zn bioavailability [56].

Soil pH typically influences the bioavailability of heavy metals and consequently their uptake by crops [57]; however, this relationship is not universal for all elements. For example, Dávid Tőzsér et al. (2023) [58] reported a significant negative correlation between soil pH and leaf Mn concentrations, but a significant positive correlation between soil pH and stem Pb concentrations in poplars, while no clear relationship was observed between pH and the tissue-specific accumulation of other metals. Many previous studies demonstrating strong pH–metal relationships were conducted under controlled laboratory conditions, often with wide pH ranges or large datasets. In contrast, our findings are based on field data, where the relatively narrow pH range may have limited the statistical significance of pH–metal correlations. A more plausible explanation in our study area is that the influence of organic matter and redox-sensitive elements (e.g., S, Se, Mn, Fe, Mo) likely overshadowed the effect of pH alone. Overall, redox-sensitive elements exert a substantial regulatory influence on heavy-metal accumulation in tuber crops. Elements such as As, Cd, and Hg exhibit predominantly negative correlations with nutrient indicators and reducing conditions (Figure 8), suggesting immobilization or fixation in nutrient-rich or strongly reducing soils. In contrast, Cu, Cr, and Zn show positive correlations with S and Mn redox activity (Figure 8), indicating enhanced bioavailability under S-rich, redox-active environments. These findings imply that adjusting soil nutrient composition and redox status under natural field conditions may help regulate the uptake of As, Cd, Cr, Cu, and other metals in tuber crops, whereas Ni and Pb appear less affected by nutrient factors and more controlled by natural soil background levels.

Considering that Cd represents the main carcinogenic risk contributor in our study area, the empirical correlations suggest that increasing soil N, P, K and S nutrients, raising Se and SOC, and elevating AN and SAK fractions while reducing A-Fe and AP fractions, may be effective management strategies to reduce Cd uptake by tuber crops under field conditions.

## 5. Conclusions

This study systematically revealed the migration characteristics and health risks of heavy metal(loid)s in tuber crops from the volcanic high background region of northern Hainan, China. The soils in this area are enriched in Cr, Cu, Ni, and Zn, reflecting a typical basalt-derived geochemical signature. Despite the strong enrichment of these elements in soils, their concentrations in tuber roots remain relatively low, indicating that metal uptake is not solely governed by total soil metal content. Health risk assessment showed that the non-carcinogenic risk (HI < 1) of heavy metal(loid)s in tubers is within the acceptable range, while Cd, Cr, and Pb are the dominant contributors to carcinogenic risk (CR > 1 × 10^−4^). The distribution of soil nutrients and available fractions plays a significant role in regulating metal bioavailability, and the abundance of redox-sensitive elements such as S, Se, Mn, and Fe strongly influences the uptake of As, Cd, Cu, and Cr by tuber crops. The volcanic high background soils display a distinct pattern of high total concentration combined with low bioavailability, suggesting that natural enrichment does not necessarily lead to crop contamination. Adjusting soil nutrient composition, particularly by increasing N, P, K, S, Se, and SOC levels while reducing available Fe and P fractions, can effectively reduce the uptake of high-risk elements such as Cd. These findings provide new insight into the migration mechanisms and health risk regulation of heavy metal(loid)s in volcanic agricultural ecosystems and offer a scientific basis for ensuring regional food security and promoting sustainable land use in naturally high background areas.

## Figures and Tables

**Table 1:** Analytical methods and detection limits for different indicators.

Indicator	Analytical Method	Detection Limit	Indicator	Analytical Method	Detection Limit
HN	VOL	1.25	As	AFS	1.00
AK	ICP-OES	1.25	Cd	ICP-MS	0.03
SAK	ICP-OES	1.25	Cr	ICP-OES	5.00
Ex-Mg	ICP-OES	1.25	Cu	ICP-MS	1.00
Ex-Na	ICP-OES	1.25	Hg	AFS	0.0005
Ex-Ca	ICP-OES	1.25	Ni	ICP-MS	2.00
A-Mn	ICP-OES	0.01	Pb	ICP-OES	2.00
A-Cu	ICP-OES	0.02	Zn	ICP-OES	4.00
A-Zn	ICP-OES	0.02	N	Combustion-GC	20.00
A-Fe	ICP-OES	0.02	P	ICP-OES	10.00
A-P	ICP-OES	0.25	K_2_O	ICP-OES	0.05%
A-Mo	ICP-MS	0.005	S	ICP-OES	20.00
Ex-Mn	ICP-OES	1.25	Se	AFS	0.01
ER-Mn	ICP-OES	1.25	SOC	HF-IR CS Analyzer	0.10%
A-S	ICP-OES	0.10	pH	Potentiometry	0.1

Note: K_2_O and SOC are expressed as percentages, while the remaining soil nutrient and trace element indicators in this table are reported in mg/kg, except for pH, which is dimensionless. Abbreviations include HN (Hydrolyzable Nitrogen), AK (Available Potassium), SAK (Slowly Available Potassium), Ex-Mg/Ex-Na/Ex-Ca/Ex-Mn (Exchangeable Mg/Na/Ca/Mn), A-Mn/A-Cu/A-Zn/A-Fe/A-P/A-Mo/A-S (Available Mn/Cu/Zn/Fe/P/Mo/S), ER-Mn (Easily Reducible Manganese), and SOC (Soil Organic Carbon).

**Table 2:** Statistical summary of soil physicochemical properties.

	pH	N	P	K_2_O	S	Se	SOC	HN	AK	SAK
Min	4.61	272	160	0.052	86.6	0.104	0.315	40.3	28.2	22.1
Max	7.72	1557	1942	0.345	1009	1.60	1.42	129	756	250
Average	5.37	809	955	0.194	441	0.761	0.809	84.3	212	91.8
CV	0.095	0.412	0.577	0.425	0.684	0.670	0.403	0.338	0.743	0.529
	Ex-Mg	Ex-Na	Ex-Ca	Ex-Mn	ERR-Mn	A-Mn	A-Cu	A-Zn	A-Fe	A-P
Min	7.86	7.36	71.5	2.18	3.75	2.55	0.361	1.04	24.9	4.20
Max	219	107	2382	146	1167	237	4.44	8.01	951	273
Average	52.6	21.2	368	40.1	345	61.5	1.82	2.91	188	91.7
CV	0.784	0.893	1.04	0.883	1.01	0.878	0.594	0.626	1.18	0.906
	A-Mo	A-S	As	Cd	Cr	Cu	Hg	Ni	Pb	Zn
Min	0.024	38.2	0.884	22.4	9.39	3.46	12.8	3.30	5.05	7.42
Max	0.635	604	6.90	151	423	75.8	312	199	30.8	159
Average	0.266	226	3.33	74.0	167	43.9	97.4	77.8	15.1	84.3
CV	0.773	0.799	0.693	0.500	0.765	0.735	0.918	0.784	0.541	0.717

Note: K_2_O and SOC are expressed in percent, Cd and Hg are expressed in μg/kg, and pH is dimensionless. All other soil nutrient and trace element indicators in this table are expressed in mg/kg.

## Data Availability

The original contributions presented in this study are included in the article. Further inquiries can be directed to the corresponding authors.
